# Analysis of selected sperm samples by a computer‐assisted system with high frame rate: A prospective study

**DOI:** 10.1002/hsr2.1217

**Published:** 2023-04-27

**Authors:** Shan‐Shan Tang, Jin‐Chun Lu, Yuan‐Hua Xu, Jing Wang, Ren‐Yun Hong, Yan‐Mei Ge, Yuan‐Jiao Liang

**Affiliations:** ^1^ Center for Reproductive Medicine, Zhongda Hospital Southeast University Nanjing Jiangsu China

**Keywords:** computer‐assisted sperm analysis, density gradient centrifugation, frame rate, selected sperm

## Abstract

**Background and Aims:**

Due to the rapid motility of the selected sperm, sperm parameters cannot be accurately determined by the manual method. So, the application of a computer‐assisted sperm analysis system with a high frame rate (HFR‐CASA, 85 Hz) in sperm selection is investigated.

**Methods:**

A total of 177 semen samples were collected for sperm selection with density gradient centrifugation. Then, the manual method and HFR‐CASA method will be used to observe and analyze the sperm concentration, motility, and percentage of progressively motile sperm (PR) of the selected sperm samples. The quality control of sperm concentration was performed with microballoons. Two selected sperm samples were analyzed 10 times repeatedly to evaluate the accuracy of the HFR‐CASA.

**Results:**

The results of microballoons analyzed with the HFR‐CASA were in control. The coefficients of variation of sperm concentration, motility, and PR from two selected sperm samples were all below 10%. The results of 177 selected sperm samples analyzed by the manual method and HFR‐CASA showed that although there were significant positive correlations in sperm concentration, motility, and PR between them (*p* < 0.001), the manual method significantly underestimated sperm concentration (*p* = 0.002) but overestimated sperm motility and PR (*p* < 0.001). When sperm concentration was below 50 × 10^6^/mL, the manual method significantly overestimated sperm concentration (*p* < 0.05). However, when sperm concentration was more than 100 × 10^6^/mL, the manual method significantly underestimated sperm concentration (*p* < 0.001). Regardless of sperm concentration, the manual method consistently overestimated sperm motility and PR (*p* < 0.001). When sperm concentration was more than 20 × 10^6^/mL, there was no correlation in sperm PR between them (*p* > 0.05). When sperm concentration was below 50 × 10^6^/mL, the correct rate of captured sperm by the HFR‐CASA was more than 98%.

**Conclusion:**

The HFR‐CASA method is more accurate than the manual method in analyzing the selected sperm samples.

## 1 INTRODUCTION

The proportion of infertile men is rising yearly because of various social, environmental, and biological factors, increasing the demand for assisted reproductive technology (ART).^1^ Sperm selection is one of the critical steps of ART. No further therapeutic scheme can be chosen without accurately detecting selected sperm suspension.^2^ However, during ART implementation, the selected sperm is mainly evaluated by the manual method. Due to the rapid motility of the selected sperm, their concentration, motility, and other relevant parameters cannot be accurately determined by the manual method using the direct observation of a microscope (Supporting Information data 1 and 2). The computer‐assisted sperm analysis (CASA) system is feasible for evaluating sperm motility.^3^ Reportedly, CASA is a more profitable method to evaluate the quality of fresh semen because of its rapid detection and ability to assess motility‐related parameters accurately.^4^ Currently, various commercial CASA systems are available. Most of them are ordinary frame rate CASA systems with frame rates between 30 and 60 Hz by the frame rates of their camera systems.^5^ The detection results of sperm moving at a low speed or in linear orbits are little affected by the frame rate of a CASA system. However, the sperm moving at a fast speed or in nonlinear orbits need to be evaluated by CASA systems which use frame rates ≥60 Hz.^6^ The selected sperm move quickly and cannot be accurately evaluated using a routine CASA system or the manual method.^5^ For this reason, the samples of selected sperm suspension were analyzed by a high frame rate CASA system (HFR‐CASA) (85 Hz) in this study. After assessing the analytical performance of the HFR‐CASA system, we further compared the results from the HFR‐CASA system and the manual method, aiming to establish an HFR‐CASA system for accurately evaluating the samples with high‐motility sperm.

## 2 MATERIALS AND METHODS

### 2.1 Source of specimens

A total of 177 outpatients treated in our center for infertility between October 2020 and November 2022 were randomly selected. After abstinence for 2–7 days, semen samples were collected from each patient by masturbation and put into an aseptic semen collection cup. After routine examination, the remaining semen samples were used in this study. This study was conducted in the Center for Reproductive Medicine of Zhongda Hospital Affiliated to Southeast University, which was approved by the Reproductive Medicine Ethics Committee of Zhongda Hospital affiliated to Southeast University (Reproduction No. 2015‐1), and all patients signed the informed consent. To demonstrate that the analysis of selected sperm should use an HFR‐CASA instead of the manual method, we performed the study.

### 2.2 Instruments and reagents

The HFR‐CASA system (GSA‐810, HARIOMED) with 85 Hz of frame rate and quality control materials for sperm concentration (Microballoon Suspense, HARIOMED, lot number: Q03‐300; nominal value: [42 ± 5] × 10^6^/mL) were provided by Hua Yue Medical Technology Co., Ltd. SpermGrad lower layer (90%), the upper layer (45%), and SpermRinse (Vitrolife) were used for sperm selection.

### 2.3 Quality control for sperm concentration

It has been reported that latex beads (microspheres or microballoons) are commonly used as quality control materials to evaluate the accuracy and stability of sperm concentration.^7^, ^8^ In this study, the microballoons were first analyzed three times repeatedly with the HFR‐CASA system to test whether the results were in control. After that, the quality control materials of microballoons were tested before each detection to ensure the results were in control.

### 2.4 Repeatability analysis

Two fresh semen samples from different patients were randomly collected, and sperm selection was performed with density gradient centrifugation (DGC). The selected sperm suspensions were detected 10 times repeatedly by the HFR‐CASA system, and the coefficient of variation (CV) was calculated.

### 2.5 Sperm selection with DGC

After routine examination, the remaining semen samples were kept at room temperature (25°C). The SpermGrad lower layer (90%), upper layer (45%), and SpermRinse solutions were taken out from a refrigerator and recovered to room temperature for further use. First, 1 mL of SpermGrad lower layer (90%) solution was added into a 15‐mL centrifuge tube. Then 1 mL of SpermGrad upper layer (45%) solution was gently added to the surface of the SpermGrad lower layer (90%) solution. Next, normally liquefied semen was slowly added to the surface of the SpermGrad lower layer (45%) solution, and a clear interface between semen and gradient solutions could be seen. After 20 min of centrifugation at 400*g* under room temperature, the upper liquids were carefully aspirated away using a pipette, and sperm sediments were transferred into a new centrifuge tube with the help of 3 mL of the SpermRinse solution. The mixture was blown up and down slowly and then centrifuged for 10 min at 200*g*. Next, the upper liquids were carefully aspirated away using a pipette, and 0.5 mL of the SpermRinse solution was added. The sperm sediments were blown gently, and the selected sperm suspension was obtained.

The obtained selected sperm suspension was divided into two portions. One portion was sent to the embryo laboratory. An embryologist with at least 5 years of working experience evaluated sperm concentration, motility, and the percentage of progressively motile sperm (PR) by the manual method. The other portion was sent to the reproductive testing laboratory, where a technician analyzed sperm concentration, motility, and PR by the HFR‐CASA system. All the detection results were sent to a third technician to analyze the data. All three technicians were blinded to each other's results.

### 2.6 Manual analysis of selected sperm suspensions

Five microliters of selected sperm suspension were loaded into a Makler counting chamber, and sperm concentration, motility, and PR were evaluated visually under an ordinary optical microscope.

### 2.7 HFR‐CASA analysis of selected sperm suspensions

**Figure 1:**
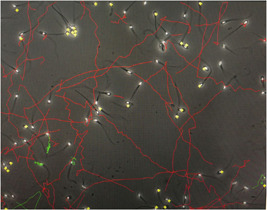
Trajectory diagram of selected sperm. Five microliters of selected sperm suspensions obtained by density gradient centrifugation were loaded into the counting chamber. Then, the counting chamber was put on the stage of a computer‐assisted sperm analysis system with a high frame rate (HFR‐CASA, 85 Hz), which was placed in a thermostated container (37°C). Subsequently, the HFR‐CASA system automatically focused and randomly selected fields of view for analysis. The trajectory diagram of the selected sperm was shown. The yellow cross represents immotile sperm. The green track represents nonprogressively motile sperm. The red track represents progressively motile sperm.

The HFR‐CASA system was equipped with a Disposable Double Chamber Slide (HARIOMED, Hua Yue Medical Technology Co., Ltd.), which consisted of one glass slide and two coverslips. Each glass slide had two counting chambers. Before adding a sample, the slide and coverslip were put on a constant temperature heating platform (37°C). First, one counting chamber was incompletely covered by one coverslip. Then, 5 μL of selected sperm suspension was loaded into the junction between the counting chamber and coverslip. Next, the coverslip was slowly pushed in parallel with the glass slide until the coverslip thoroughly covered the counting chamber. Last, the counting chamber was put on the stage of the HFR‐CASA system, which was placed in a thermostated container (37°C). In analyzing selected sperm suspension, 15 fields of view were selected from each sample randomly, and each field of view was scanned at 85 frames per second. The first 50 frames of pictures for each sample field were saved. Finally, the results of sperm concentration, motility, and PR were outputted automatically by the HFR‐CASA system. The total analysis time of each sample was about 1 min. The trajectory diagram of the selected sperm is shown in Figure 1.

### 2.8 Analysis of correct rates for captured sperm by HFR‐CASA

**Figure 2:**
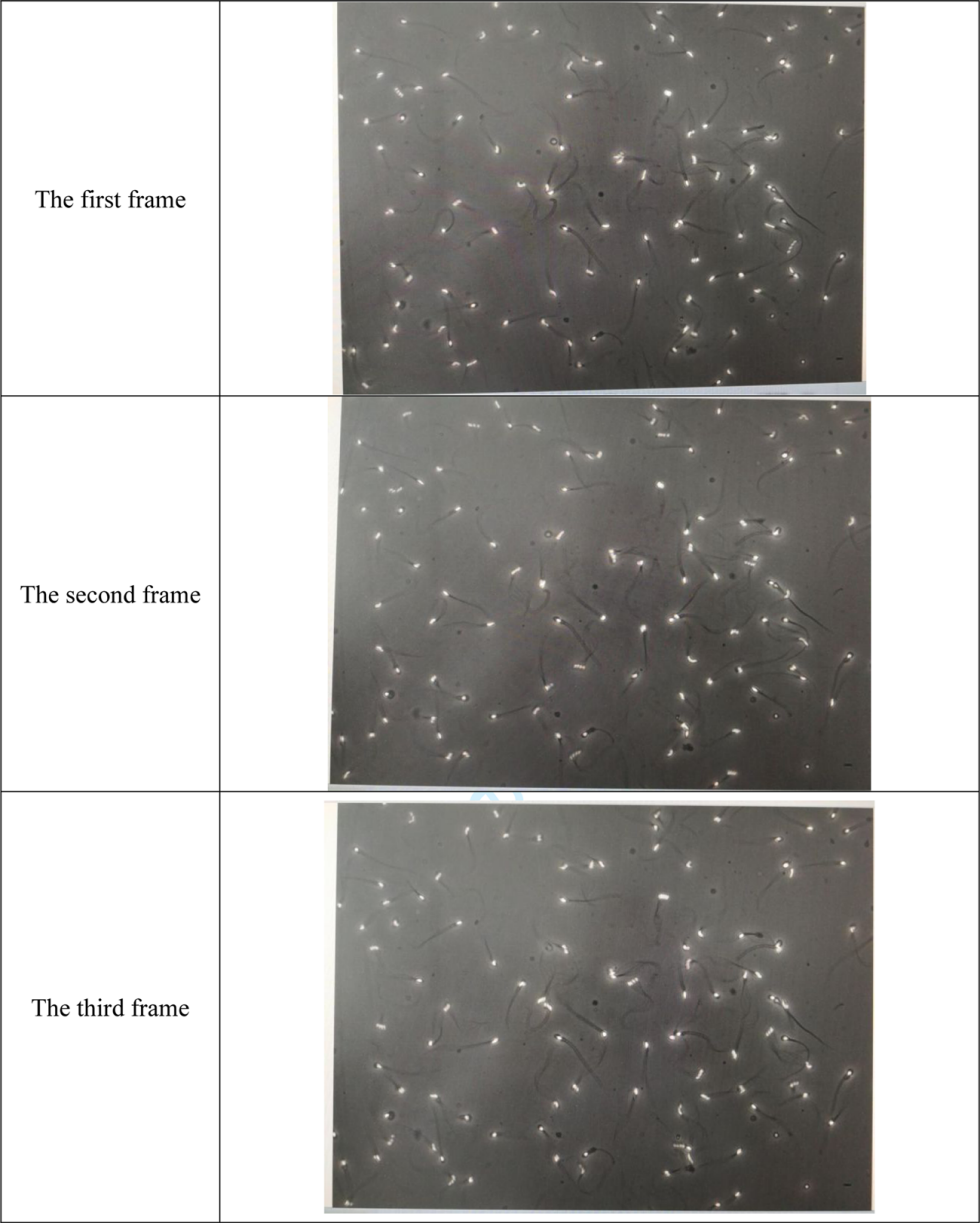
Pictures of the first, second, and third frames for the same field of view. Five microliters of selected sperm suspensions obtained by density gradient centrifugation were loaded into the counting chamber. Then, the counting chamber was put on the stage of a computer‐assisted sperm analysis system with a high frame rate (HFR‐CASA, 85 Hz), which was placed in a thermostated container (37°C). Subsequently, the HFR‐CASA system automatically focused and randomly selected fields of view for analysis. Fifteen fields of view were selected from each sample with selected sperm randomly, and each field of view was scanned at 85 frames per second. The first 50 frames of pictures for each sample field were saved. By observing the pictures of the first, second, and third frames consecutively, sperm and impurities could be differentiated obviously, and thereby the correct rates of captured sperm by the HFR‐CASA system could be determined.

The sperm concentration of a sample was determined based on sperm in the first frame of each field counted by the HFR‐CASA system. So, the manual validation of correct rates for captured sperm by the HFR‐CASA system was also conducted as sperm in the first frame of each field. By observing the pictures of the first, second, and third frames consecutively (Figure 2), sperm and impurities could be differentiated obviously, and thereby the correct rates of captured sperm by the HFR‐CASA system could be determined. The sperm pictures from six samples of selected sperm suspension at different concentrations were selected randomly, and the numbers of sperm in the foremost three frames of each field were validated manually. Then, the manual validation results were compared with the counting results by the HFR‐CASA system. The correct rate of captured sperm was calculated as numbers of sperm counted by the HFR‐CASA system/numbers of sperm validated manually ×100%.

### 2.9 Statistical analysis

The data were analyzed by statistical software SPSS 20.0 (SPSS Inc.). The accuracy (repeatability) assessment data of the HFR‐CASA system were expressed as mean ± standard deviation (SD), and their CVs were calculated. The data of sperm concentration, motility, and PR for selected sperm suspensions were first performed by one‐sample nonparametric tests (Kolmogorov–Smirnov test) to determine whether they were normal distribution. The data conforming to normal distribution were expressed as mean ± SD, and those conforming to nonnormal distribution were expressed as median [P25, P75]. For the comparisons and hierarchical comparisons of sperm concentration, motility, and PR for selected sperm suspensions between the manual method and HFR‐CASA system, if the data conform to a normal distribution, paired *t* test was used; if the data conform to a nonnormal distribution, Wilcoson‐signed rank test was used. For the correlations of sperm concentration, motility, and PR for selected sperm suspensions between the manual method and HFR‐CASA system, when the data were consistent with normal distribution, Pearson correlation analysis was conducted. Otherwise, Spearman correlation analysis was done. They all performed the two‐sided test. *p* ≤ 0.05 was considered to be statistically significant.

## 3 RESULTS

### 3.1 Analysis results of microballoons

The quality control materials of microballoons with (42 ± 5) × 10^6^/mL of nominal value were detected three times by the HFR‐CASA system repeatedly. The results were 40.5, 39.9, and 40.1 × 10^6^/mL, all of which were in the range of the nominal value. The results of microballoons before each subsequent detection were also in the nominal value range.

### 3.2 Accuracy assessment of the HFR‐CASA system

The CVs of sperm concentration, motility, and PR from two samples of selected sperm suspension analyzed 10 times by the HFR‐CASA system were below 10% (Table 1).

**Table 1:** Accuracy assessment of the HFR‐CASA system.

Sperm parameters	*n*	Sample 1	CV (%)	Sample 2	CV (%)
Concentration (×10^6^/mL)	10	46.11 ± 3.01	6.52	81.23 ± 6.20	7.63
Motility (%)	10	95.87 ± 5.03	5.25	97.63 ± 3.15	3.22
PR (%)	10	77.76 ± 6.70	8.61	81.16 ± 4.46	5.50

*Note*: The data were expressed as mean ± standard deviation (SD).

Abbreviations: CV, coefficient of variation; HFR‐CASA, computer‐assisted sperm analysis system with high frame rate; PR, percentage of progressively motile sperm.

### 3.3 Comparisons and correlations of the results for selected sperm suspensions between the manual method and the HFR‐CASA system

A total of 177 samples of selected sperm suspension were analyzed by the manual method and the HFR‐CASA system. The results showed that sperm concentration, motility, and PR were all significantly and positively correlated (*r* = 0.849, 0.502, and 0.404, *p* < 0.001). Compared with the HFR‐CASA system, the manual method significantly underestimated sperm concentration (*p* = 0.002) but overestimated sperm motility and PR (*p* < 0.001, Table 2).

**Table 2:** Comparisons and correlations of the results for selected sperm suspensions between the manual method and HFR‐CASA system.

Sperm parameters	*n*	Manual method	HFR‐CASA	*Z/P*	*r/P*
Concentration (×10^6^/mL)	177	45 [20, 92.5]	44.8 [13.65, 106.35]	−3.156/0.002	0.849/<0.001
Motility (%)	177	95 [85, 95]	79.3 [65.45, 86.7]	−9.899/<0.001	0.502/<0.001
PR (%)	177	80 [76.5, 90]	64.6 [51.8, 74.55]	−9.969/<0.001	0.404/<0.001

*Note*: The data conformed to nonnormal distribution and were expressed as median [P25, P75]. Wilcoson‐signed rank test was used to compare the results of sperm concentration, motility, and PR for selected sperm suspensions between the manual method and the HFR‐CASA system. Spearman correlation analysis was used to analyze the correlations of sperm concentration, motility, and PR for selected sperm suspensions between the manual method and the HFR‐CASA system. Although sperm concentration, motility, and PR detected by the manual method were significantly and positively correlated with those by the HFR‐CASA system (*p* < 0.001), the manual method significantly underestimated sperm concentration (*p* = 0.002) but overestimated sperm motility and PR (*p* < 0.001).

Abbreviations: HFR‐CASA, computer‐assisted sperm analysis system with high frame rate; PR, percentage of progressively motile sperm.

### 3.4 Hierarchical comparisons of the results for selected sperm suspensions between the manual method and the HFR‐CASA system

High sperm concentration might affect the analysis results of sperm motility. So, we compared the differences and correlations of sperm concentration, motility, and PR between the manual method and the HFR‐CASA system hierarchically according to sperm concentrations detected by the HFR‐CASA system, and the results were shown in Tables 3, 4, 5, 6. When sperm concentration was below 50 × 10^6^/mL, the manual method significantly overestimated sperm concentration (*p* < 0.05). However, when sperm concentration was more than 100 × 10^6^/mL, the manual method significantly underestimated sperm concentration (*p* < 0.001). When sperm concentration was 50.1–99.9 × 10^6^/mL, there was no significant difference in sperm concentration between the two methods (*p* > 0.05). Regardless of sperm concentration, the manual method always overestimated sperm motility and PR (*p* < 0.001). There was a significant correlation in sperm PR between the two methods only when sperm concentration was ≤20 × 10^6^/mL. When sperm concentration was more than 20 × 10^6^/mL, there was no correlation in sperm PR between the two methods (*p* > 0.05). Regardless of sperm concentration, sperm concentration detected by the manual method was significantly and positively correlated with that by the HFR‐CASA system (*p* < 0.01). When sperm concentration was <100 × 10^6^/mL, there was a significant correlation in sperm motility between the two methods. However, when sperm concentration was ≥100 × 10^6^/mL, there was no correlation in sperm motility between them (*p* = 0.496).

**Table 3:** Comparisons and correlations of results between the manual method and HFR‐CASA system (sperm concentration ≤20 × 10^6^/mL).

Sperm parameters	*n*	Manual method	HFR‐CASA	*Z/P*	*r/P*
Concentration (×10^6^/mL)	55	10 [8, 25]	10.1 [6.8, 13]	−2.065/0.04	0.478/<0.001
Motility (%)	55	80 [60, 95]	60.6 [46.9, 75.9]	−4.671/<0.001	0.703/<0.001
PR (%)	55	70 [40, 85]	54.7 [41, 66.4]	−4.106/<0.001	0.590/<0.001

*Note*: The data conformed to nonnormal distribution and were expressed as median [P25, P75]. Wilcoson‐signed rank test was used to compare the results of sperm concentration, motility, and PR for selected sperm suspensions between the manual method and the HFR‐CASA system. Spearman correlation analysis was used to analyze the correlations of sperm concentration, motility, and PR for selected sperm suspensions between the manual method and the HFR‐CASA system. When sperm concentration was below 20 × 10^6^/mL, although sperm concentration, motility, and PR detected by the manual method were significantly and positively correlated with those by the HFR‐CASA system (*p* < 0.001), the manual method significantly overestimated sperm concentration, motility, and PR (*p* < 0.05 or 0.001).

Abbreviations: HFR‐CASA, computer‐assisted sperm analysis system with high frame rate; PR, percentage of progressively motile sperm.

**Table 4:** Comparisons and correlations of results between the manual method and HFR‐CASA system (sperm concentration: 20.1−50 × 10^6^/mL).

Sperm parameters	*n*	Manual method	HFR‐CASA	*t* or *Z/P*	*r/P*
Concentration (×10^6^/mL)	39	47.38 ± 26.10	34.66 ± 8.59	−3.380/0.002	0.452/0.004
Motility (%)	39	95 [90, 95]	78.8 [69.7, 84.7]	−4.933/<0.001	0.323/0.05
PR (%)	39	85 [80, 90]	67.1 [58.5, 74.1]	−4.982/<0.001	0.239/0.14

*Note*: Sperm concentration conformed to the normal distribution and was expressed as mean ± SD. Sperm motility and PR conformed to nonnormal distribution and were expressed as median [P25, P75]. Paired *t* test was used to compare the results of sperm concentration for selected sperm suspensions between the manual method and the HFR‐CASA system, while Wilcoson‐signed rank test was used to compare the results of sperm motility and PR. Pearson correlation analysis was used to analyze the correlations of sperm concentration for selected sperm suspensions between the manual method and the HFR‐CASA system, while Spearman correlation analysis was used to analyze the correlations of sperm motility and PR. When sperm concentration was 20.1−50 × 10^6^/mL, the manual method significantly overestimated sperm concentration, motility, and PR (*p* < 0.05 or 0.001). There were significant correlations in sperm concentration and motility between the two methods (*p* < 0.05), but there was no correlation in sperm PR between them (*p* > 0.05).

Abbreviations: HFR‐CASA, computer‐assisted sperm analysis system with high frame rate; PR, percentage of progressively motile sperm.

**Table 5:** Comparisons and correlations of results between the manual method and HFR‐CASA system (sperm concentration: 50.1−99.9 × 10^6^/mL).

Sperm parameters	*n*	Manual method	HFR‐CASA	*Z/P*	*r/P*
Concentration (×10^6^/mL)	38	69 [34.25, 85]	69.95 [56.28, 85]	−1.769/0.08	0.419/0.009
Motility (%)	38	95 [92.75, 95]	83.95 [78.4, 88.58]	−5.373/<0.001	0.324/0.05
PR (%)	38	85 [80, 88.5]	69.4 [61.18, 77.35]	−5.199/<0.001	−0.043/0.80

*Note*: The data conformed to nonnormal distribution and were expressed as median [P25, P75]. Wilcoson‐signed rank test was used to compare the results of sperm concentration, motility, and PR for selected sperm suspensions between the manual method and the HFR‐CASA system. Spearman correlation analysis was used to analyze the correlations of sperm concentration, motility, and PR for selected sperm suspensions between the manual method and the HFR‐CASA system. When sperm concentration was 50.1–99.9 × 10^6^/mL, there was no significant difference in sperm concentration between the two methods (*p* > 0.05), but sperm motility and PR detected by the HFR‐CASA system were significantly lower than those by the manual method (*p* < 0.001). There were significant correlations in sperm concentration and motility between the two methods (*p* < 0.05), but there was no correlation in sperm PR between them (*p* > 0.05).

Abbreviations: HFR‐CASA, computer‐assisted sperm analysis system with high frame rate; PR, percentage of progressively motile sperm.

**Table 6:** Comparisons and correlations of results between the manual method and HFR‐CASA system (sperm concentration ≥ 100 × 10^6^/mL).

Sperm parameters	*n*	Manual method	HFR‐CASA	*t* or *Z/P*	*r/P*
Concentration (×10^6^/mL)	45	115 ± 36.02	177.68 ± 50.29	10.574/<0.001	0.620/<0.001
Motility (%)	45	95 [90, 95]	85.2 [77.4, 91.45]	−5.232/<0.001	−0.104/0.50
PR (%)	45	85 [80, 90]	68.7 [55.1, 75.6]	−5.841/<0.001	0.284/0.06

*Note*: Sperm concentration conformed to the normal distribution and was expressed as mean ± SD. Sperm motility and PR conformed to nonnormal distribution and were expressed as median [P25, P75]. Paired *t* test was used to compare the results of sperm concentration for selected sperm suspensions between the manual method and the HFR‐CASA system, while Wilcoson‐signed rank test was used to compare the results of sperm motility and PR. Pearson correlation analysis was used to analyze the correlations of sperm concentration for selected sperm suspensions between the manual method and the HFR‐CASA system, while Spearman correlation analysis was used to analyze the correlations of sperm motility and PR. When sperm concentration was ≥100 × 10^6^/mL, the manual method significantly underestimated sperm concentration but overestimated sperm motility and PR (*p* < 0.001). There were significant correlations in sperm concentration between the two methods (*p* < 0.001), but there was no correlation in sperm motility and PR between them (*p* > 0.05).

Abbreviations: HFR‐CASA, computer‐assisted sperm analysis system with high frame rate; PR, percentage of progressively motile sperm.

### 3.5 Analysis of correct rates for captured sperm by the HFR‐CASA system

The results of manual validation for six samples of selected sperm suspension at different sperm concentrations showed no significant difference in the numbers of sperm counted by the manual validation and HFR‐CASA system (*p* > 0.05, Table 7). When sperm concentration was below 50 × 10^6^/mL, the correct rate of captured sperm by the HFR‐CASA system was more than 98%. With the increase in sperm concentration, the correct rate of captured sperm by the HFR‐CASA system declined, but they were within (100 ± 6)%.

**Table 7:** Correct rates of captured sperm by the HFR‐CASA system.

No. of sample	Sperm concentration (×10^6^/mL)	Number of fields	HFR‐CASA	Manual validation	*p*	Correct rates of captured sperm (%)
20,201,104,002	17.5	13	16.92 ± 3.20	17.23 ± 3.54	0.34	98.2 (220/224)
20,201,023,002	36	8	35.13 ± 2.75	35.25 ± 2.96	0.89	99.6 (281/282)
20,201,126,004	51.8	11	50.09 ± 4.41	50.27 ± 3.88	0.62	99.6 (551/553)
20,201,222,002	65.4	5	65.0 ± 4.36	62.6 ± 3.58	0.05	103.8 (325/313)
20,201,102,003	100.5	7	99.0 ± 1.83	99.29 ± 1.89	0.74	99.7 (693/695)
20,201,105,001	138.0	3	138.0 ± 10.82	130.33 ± 7.02	0.11	105.9 (414/391)

*Note*: The sperm pictures from six samples of selected sperm suspension at different concentrations were selected randomly, and the numbers of sperm in the foremost three frames of each field were validated manually. The correct rate of captured sperm was calculated as numbers of sperm counted by the HFR‐CASA system/numbers of sperm validated manually ×100%.

Abbreviations: HFR‐CASA, computer‐assisted sperm analysis system with high frame rate; PR, percentage of progressively motile sperm.

## 4 DISCUSSION

Male factors account for 20%–30% of infertile couples.^9^ An increasing number of infertile couples are seeking the help of ART. Sperm selection is one of the critical steps of ART.^10^ The infertile couples can select appropriate ART according to the total number of progressive motile sperm after selection, such as intrauterine insemination (IUI), in vitro fertilization and embryo transfer (IVF‐ET), or intracytoplasmic sperm injection (ICSI).^11^, ^12^ Therefore, accurate analysis of sperm concentration, motility, and especially PR after selection is very important for the clinical selection of appropriate ART and assessment of therapeutic effects.

At present, most reproductive medicine centers have adopted the manual method to evaluate the selected sperm suspension in China. However, the manual method largely depended on the experience of a technician and thus was very subjective, which would lead to considerable variation in the evaluation results among technicians. Moreover, the moving speed of the selected sperm was very rapid, and the manual method could not evaluate their sperm motility and concentration accurately, especially in the selected sperm suspension with high sperm concentration. The CASA system has been extensively applied in routine semen analysis of andrology laboratories. The CASA system was more objective, accurate, and rapid than the manual method and was especially suitable for the analysis of sperm motility.^13^ However, the detection results of an ordinary CASA system for a sample with high‐motility sperm were not ideal owing to the collision between sperm and the low frame rate of the CASA system.^14^ Whether an HFR‐CASA system could obtain accurate results for such samples? There was little research on this. Therefore, we first assessed the analytical performance of the HFR‐CASA system, and further compared the results of the selected sperm suspensions detected by the manual method and HFR‐CASA system, respectively.

We first evaluated the accuracy and repeatability of the HFR‐CASA system. Quality control materials of microballoons were used to ensure the detection accuracy of sperm concentration. The quality control results of repeated detection three times and the subsequent detection were in the nominal value range, indicating that the HFR‐CASA system could ensure the analysis accuracy of sperm concentration. The CVs of sperm concentration, motility, and PR from two samples of selected sperm suspension detected by the HFR‐CASA system were below 10%, indicating that the HFR‐CASA system had good repeatability for the analysis of sperm concentration and motility. The manual validation for the correct rates of captured sperm in six samples of selected sperm suspension at different sperm concentrations showed no significant difference in the number of captured sperm between the HFR‐CASA system and manual validation (*p* > 0.05). When sperm concentration was below 50 × 10^6^/mL, the correct rates of captured sperm by the HFR‐CASA system were more than 98%. As sperm concentration increased, the correct rates of captured sperm by the HFR‐CASA system declined but were within (100 ± 6)%, indicating that the correct rates of captured sperm by the HFR‐CASA system were more than 94% even in analyzing the samples with high sperm concentration (>100 × 10^6^/mL), and that the HFR‐CASA system could accurately analyze the samples with high sperm concentration without dilution.

The analysis results of 177 samples of selected sperm suspension showed that although sperm concentration, motility, and PR were all significantly and positively correlated between the two methods (*p* < 0.001), the manual method significantly overestimated sperm motility and PR (*p* < 0.001) but underestimated sperm concentration (*p* = 0.002), indicating that the manual method was unsuitable for evaluating the samples with high‐motility sperm. The results of sperm concentration, motility, and PR detected by the two methods were further analyzed hierarchically, and it was found that there was a significant correlation in sperm PR between the two methods only when sperm concentration was ≤20 × 10^6^/mL. When sperm concentration was more than 20 × 10^6^/mL, there was no correlation in sperm PR between the two methods (*p* > 0.05). When sperm concentration was below 50 × 10^6^/mL, the manual method significantly overestimated sperm concentration (*p* < 0.05). However, when sperm concentration was more than 100 × 10^6^/mL, the manual method significantly underestimated sperm concentration (*p* < 0.001). Regardless of sperm concentration, the manual method always overestimated sperm motility and PR (*p* < 0.001). All of these indicated that the manual method was unsuitable for analyzing sperm concentration, motility, and PR of the samples with high‐motility sperm regardless of sperm concentration and that an HFR‐CASA system might be the only choice at present.

Although it was reported that the use of a CASA system for routine semen analysis was not suggested in samples with sperm concentration <15 × 10^6^ or >100 × 10^6^/mL,^13^ our recent research on 3 samples with sperm concentrations about 100 × 10^6^/mL diluted into 50, 25, 15, 10, 5, and 2 × 10^6^/mL with their own seminal plasma showed that the *R* ^2^ value of sperm concentration within the range of 2–100 × 10^6^/mL for each sample detected by a CASA system with a frame rate of 50 Hz was ≥0.99, suggesting that the sperm concentration of a sample with sperm concentration <15 × 10^6^/mL could be detected accurately by an ordinary CASA system. Moreover, we found that the accuracy of sperm motility or PR had little to do with sperm concentration and mainly depended on sperm motility or PR values (unpublished data). Since the number of samples with sperm concentration >100 × 10^6^/mL was relatively small and such samples were considered normal, it was of little significance to study these samples. Therefore, we could think that the HFR‐CASA system was not only suitable for the analysis of selected sperm samples but also for routine semen analysis.

The frame rate may affect sperm motion parameters. It was reported that completely different results could be obtained only by changing the frame rate.^15^ The CASA system with a frame rate of 25–50 Hz could provide valuable human sperm motion parameters, but the results obtained by different frame rate settings were not comparable. For human sperm, 60 Hz was the minimum imaging frequency standard for reliable analysis.^16^ A CASA system could work extremely well on washed human sperm populations, which usually had very high motility and minimal contamination of other cells and debris.^17^ Therefore, the HFR‐CASA system with a frame rate of 85 Hz used in this study might also be suitable for analyzing sperm motion parameters.

It was reported that a CASA system with a frame rate of 15 Hz could be a reliable diagnostic tool for routine semen analysis, which could provide clinically acceptable results based on the 5th edition of the World Health Organization (WHO) guidelines.^18^ If the error were corrected, a CASA system with a frame rate of 25 Hz would produce fast, accurate, and precise results with better analytical variance than the manual method.^19^ Vernon et al.^20^ conducted a study to determine the precision and accuracy of the Accu‐Beads and their utility as a quality control product for sperm concentration of the manual method and CASA system with a frame rate of 25 Hz, and found that the CASA system produced more reproducible results than the manual method. Komori et al.^21^ found a strong correlation between the CASA and manual method results. Freour et al.^22^ pointed out that the key to CASA performance was quality control rather than a strict correlation with the manual semen analysis. Therefore, on the basis of quality control, the HFR‐CASA system used in this study should have a better application prospect than the existing ordinary CASA systems.

Nevertheless, three considerations should be paid attention to when using the HFR‐CASA system to analyze the samples with high‐motility sperm. First, the counting chambers should be used correctly. The standard sperm counting chambers include reusable counting chambers, such as Makler and disposable counting chambers, such as Leja.^23^ In this study, disposable counting chambers were used. Regardless of reusable or disposable counting chambers, the loading quantity should be correct, and the loaded sample volume should be filled with the gaps of a counting chamber. In this study, the loading quantity of each sample was 5 μL. After loading, the counting chamber should be placed for 10 s until the liquid stabilizes, which could prevent the fields of view from drifting or shaking during analysis. In addition, bubbles should be avoided during loading. It was reported that reusable sperm counting chambers could make sperm distribution in the chamber more uniform, and the counting results were more accurate.^24^ Whether the provision of reusable sperm counting chambers in the HFR‐CASA system will further improve the analytical performance deserves further study. Second, the threshold values judging nonprogressive sperm (NP) and immotile sperm (IM) in an HFR‐CASA system should be appropriately raised. In the samples with high‐motility sperm, NP and IM sperm are easily impacted by rapid‐moving sperm, thus leading to a slight shift. If the threshold values of NP and IM sperm in the HFR‐CASA system are set as an ordinary CASA system, NP and IM sperm may be misjudged as PR sperm. Third, the effect of sperm concentration should be considered. When sperm concentration is more than 50 × 10^6^/mL, the collision between sperm may affect the movement orbits of sperm, leading to the fluctuation in the results of sperm concentration. So, before the samples with high‐motility sperm are analyzed in the course of daily laboratory work, sperm concentrations should be diluted to be below 50 × 10^6^/mL. The WHO manual (sixth) recommends that semen samples with high sperm concentration should be diluted with seminal plasma from the same patient, since dilution with normal saline may affect sperm motility.^25^ For the selected samples with high‐motility sperm, we think that a special sperm nutrient solution may be more appropriate.

## 5 CONCLUSIONS

The HFR‐CASA system used in this study may accurately analyze sperm concentration, motility, and PR of selected sperm suspensions, but the manual method overestimates sperm motility, and PR. Moreover, the manual method significantly overestimated sperm concentration for the samples with low sperm concentration but underestimated sperm concentration for the samples with high sperm concentration. Therefore, the samples with selected sperm suspension can be analyzed by an HFR‐CASA system. The accurate analysis of selected sperm suspensions by an HFR‐CASA system may lay the foundations for clarifying the relationships of the total number of selected PR sperm with the selection of IUI, IVF‐ET, or ICSI and pregnancy outcomes of IUI, IVF‐ET, and ICSI. Moreover, an HFR‐CASA system may be used to compare the effect of sperm selection of different sperm selection methods, such as DGC, microfluidic technology, swim‐up method, glass fiber filtration, serum albumin filtration, and swimming precipitation^26^ Nevertheless, the specific operational details of the HFR‐CASA system for analyzing the samples with high‐motility sperm need to be standardized and trained.

## AUTHOR CONTRIBUTIONS

*Conception and design*: Jin‐Chun Lu. *Administrative support*: Yuan‐Jiao Liang. *Provision of study materials or patients*: Jin‐Chun Lu and Yuan‐Jiao Liang. *Collection and assembly of data*: Shan‐Shan Tang, Yuan‐Hua Xu, Jing Wang, Ren‐Yun Hong and Yan‐Mei Ge. *Data analysis and interpretation*: Jin‐Chun Lu and Shan‐Shan Tang. *Manuscript writing*: All authors. *Final approval of manuscript*: All authors. All authors have read and approved the final version of the manuscript, full access to all of the data in this study and takes complete responsibility for the integrity of the data and the accuracy of the data analysis.

## CONFLICT OF INTEREST STATEMENT

The authors declare no conflict of interest.

## TRANSPARENCY STATEMENT

The lead author Jin‐Chun Lu affirms that this manuscript is an honest, accurate, and transparent account of the study being reported; that no important aspects of the study have been omitted; and that any discrepancies from the study as planned (and, if relevant, registered) have been explained.

## Supporting information


**Supplementary video 1: Motion video of selected sperm**. Selected sperm were obtained by density gradient centrifugation. Five microliters of selected sperm suspensions were loaded into the counting chamber. Then, the counting chamber was put on the stage of a computer‐assisted sperm analysis system with a high frame rate (HFR‐CASA, 85 Hz), which was placed in a thermostated container (37℃). Subsequently, the HFR‐CASA system automatically focused and recorded sperm motion videos. The sperm movement speed shown in this video is relatively slow, and the manual method can still count the number of sperm.

**Supplementary video 2: Motion video of selected sperm**. Selected sperm were obtained by density gradient centrifugation. Five microliters of selected sperm suspensions were loaded into the counting chamber. Then, the counting chamber was put on the stage of a computer‐assisted sperm analysis system with a high frame rate (HFR‐CASA, 85 Hz), which was placed in a thermostated container (37℃). Subsequently, the HFR‐CASA system automatically focused and recorded sperm motion videos. The sperm movement speed shown in this video is very fast, and it is impossible to accurately count the number of sperm using the manual method.

## Data Availability

The data are available from the corresponding author upon reasonable request.
